# Benefits of dietary polyphenols in Alzheimer’s disease

**DOI:** 10.3389/fnagi.2022.1019942

**Published:** 2022-12-13

**Authors:** Farida El Gaamouch, Fiona Chen, Lap Ho, Hsiao-Yun Lin, Chongzhen Yuan, Jean Wong, Jun Wang

**Affiliations:** ^1^Department of Neurology, Icahn School of Medicine at Mount Sinai, New York, NY, United States; ^2^Geriatric Research, Education and Clinical Center, James J Peters VA Medical Center, Research & Development, Bronx, NY, United States; ^3^Department of Genetics and Genomic sciences, Icahn School of Medicine at Mount Sinai, New York, NY, United States

**Keywords:** polyphenol, Alzheimer, oxidative stress, neuroinflammation, synaptic plasticity

## Abstract

Alzheimer′s disease (AD) is an irreversible progressive neurodegenerative disease affecting approximately 50 million people worldwide. It is estimated to reach 152 million by the year 2050. AD is the fifth leading cause of death among Americans age 65 and older. In spite of the significant burden the disease imposes upon patients, their families, our society, and our healthcare system, there is currently no cure for AD. The existing approved therapies only temporarily alleviate some of the disease’s symptoms, but are unable to modulate the onset and/or progression of the disease. Our failure in developing a cure for AD is attributable, in part, to the multifactorial complexity underlying AD pathophysiology. Nonetheless, the lack of successful pharmacological approaches has led to the consideration of alternative strategies that may help delay the onset and progression of AD. There is increasing recognition that certain dietary and nutrition factors may play important roles in protecting against select key AD pathologies. Consistent with this, select nutraceuticals and phytochemical compounds have demonstrated anti-amyloidogenic, antioxidative, anti-inflammatory, and neurotrophic properties and as such, could serve as lead candidates for further novel AD therapeutic developments. Here we summarize some of the more promising dietary phytochemicals, particularly polyphenols that have been shown to positively modulate some of the important AD pathogenesis aspects, such as reducing β-amyloid plaques and neurofibrillary tangles formation, AD-induced oxidative stress, neuroinflammation, and synapse loss. We also discuss the recent development of potential contribution of gut microbiome in dietary polyphenol function.

## Introduction

AD is a complex disease, which makes its pathophysiology difficult to decipher and consequently challenging to treat or cure. The classical pathologic hallmarks of AD include extracellular accumulation of β-amyloid (Aβ) and intracellular Tau protein aggregation which lead to, respectively, neuritic plaques and neurofibrillary tangles’ formation in the brain. The amyloid plaques and tau tangles aggregate trigger successive deleterious events and other chronic aberrant central nervous system (CNS) features such as hyperactive inflammation, oxidative stress, and sub-optimal energy metabolism which eventually lead to synapse loss and neuronal death. These cellular damages manifest in patients as a progressive neurocognitive impairment accompanied by language alterations and a progressive deterioration of a person’s ability to perform everyday activities (Alzheimers Dement, 2020).

Aging is the greatest risk factor for AD. Many genetic risk factors have also been identified, with ApoE4 being the biggest known genetic risk factor. However, there are also lifestyle and environmental risk factors for AD, including lifestyle conditions associated with diabetes and cardiovascular diseases and environmental factors leading to traumatic brain injuries and depression (Armstrong, 2019). Aging and, to a large extent, genetic risk factors, are not amenable to modification (Riedel et al., 2016). In contrast, lifestyle and environmental risks are more readily modifiable. Moreover, there is increasing evidence implicating specific lifestyle factors (e.g., dietary factors such as specific phytochemicals) or environmental factors (e.g., exercise) may protect against AD mechanisms (Xu et al., 2015; Dominguez et al., 2021; Guasch-Ferré and Willett, 2021; Zhang et al., 2021). There is increasing interest in novel AD treatments targeting select relevant lifestyles or environmental factors. In this review, we will focus our discussion on a specific subclass of protective dietary phytochemicals, namely polyphenols, with promise for AD therapeutics.

Dietary components have a direct molecular impact on AD. Over the past decade, a widely distributed subclass of dietary components, polyphenols, have raised great interest in the scientific community for their potential role in protection against AD. Substantial number of clinical trials have been conducted to assess their clinical benefits against AD and associated cognitive impairments using diverse source of polyphenols: either in the form of whole fruit or fruit products such as blueberry, grape juice, pomegranate juice (Krikorian et al., 2010; Krikorian et al., 2012; Bookheimer et al., 2013; Krikorian et al., 2022), or in the form of extracts such as curcumin, grape seed polyphenol extract (GSPE), or pure synthetic material such as resveratrol (Baum et al., 2008; Kennedy et al., 2010; Patel et al., 2011; Mecocci and Polidori, 2012; Ringman et al., 2012; Turner et al., 2015; Moussa et al., 2017). The historical interest in dietary polyphenols is attributed to their high abundance in general food supplies and their antioxidant properties (Scalbert et al., 2005). However, current research of the benefits of dietary polyphenols is largely focused on their interaction and modulation of metabolic pathways regulating inflammation (Maleki et al., 2019; Ansari et al., 2020), endothelial function (Patel et al., 2018; Li et al., 2019; Parsamanesh et al., 2021), fatty acids, amino acids and carbohydrates metabolism (Hanhineva et al., 2010; Wang S et al., 2014; Naveed et al., 2018; Rothenberg et al., 2018; Różańska and Regulska-Ilow, 2018). Collectively, polyphenols’ ability to block free radicals’ activity, repair DNA damage, modulate the gene expression involved in metabolism, and act as signaling molecules to promote antioxidant defense support the development of dietary polyphenols in AD and other diseases (Azqueta and Collins, 2016; Hussain et al., 2016; Jiang, 2019; Maleki et al., 2019; Prasanth et al., 2019; Xing et al., 2019; Ohishi et al., 2021; Shen et al., 2022).

In this review, we summarize the major molecular mechanisms that correlate the health benefits of dietary polyphenols in AD physiopathology, focusing on the potential effects of these polyphenols to protect against Tau- and Aβ-mediated pathogenesis, oxidative stress, inflammation, synapse loss and memory deterioration.

## Polyphenols

Polyphenols are a class of organic compounds characterized by the presence of more than one phenol structural unit (several hydroxyl groups on aromatic rings). These phytochemicals have a protective role in plants involving in defense against ultraviolet radiation or pathogens invasion. They are mainly found in plant-based food diet (Manach et al., 2004; Maraldi et al., 2014). The number of phenol rings in their molecular structure will define the chemical subclass they belong to. More than 8,000 naturally occurring polyphenols exist and can be grouped in 4 chemical subclasses: flavonoids, phenolic acids, stilbenes, and lignans (Manach et al., 2004; Maraldi et al., 2014).

### Flavonoids

Flavonoids are the largest and most widespread groups of plant-derived secondary metabolites, with a 15-carbon skeleton, that have been described to exert beneficial effects in the prevention of neurodegenerative diseases (Dai et al., 2006; Kuriyama et al., 2006). Their highly reactive hydroxyl group is largely responsible for their ability to scavenge free radicals and/or chelate metal ions (Kumar et al., 2013; Kumar and Pandey, 2013). Flavonoids can be subdivided into different subgroups: Flavonols, with quercetin and kaempferaol as the representative compounds, are found in all types of food, with higher quantity in onions, broccoli, kale, blueberries and red wine (Herrmann, 1976). In comparison, flavones (luteolin and apigenin) are much less present in fruits and vegetables. Isoflavones are phytoestrogens mainly found in legume such as soya (Coward et al., 1993; Reinli and Block, 1996). Catechin and epicatechin are the basic units of flavanols and they form various oligomers and polymers through C4-C8 or C4-C6 interflavan bonds. Flavanols are found in many types of fruit and in red wine, however green tea and chocolate are the richest sources (Lakenbrink et al., 2000). Anthocyanins (cyanidin, malvidin, etc.) and chalocone (phloretin, arbutin, etc.) are mostly abundant in fruits and vegetables.

### Phenolic acids

Phenolic acids are the most abundant group of bioactive compounds present in almost all plants (Rashmi and Negi, 2020). Phenolic acids are hydroxyl derivatives of benzoic and cinnamic acid. Hydroxybenzoic acid is found in very low quantity in plants. Hydroxycinnamic acids mainly consist of coumaric, sinapic, caffeic and ferulic acids. The richest dietary source of hydroxycinnamic acids are cherries, apples, berries and kiwi (Fleuriet et al., 1990). Caffeic acid is the most abundant hydroxycinnamic acid in many fruits and ferulic acid is mostly found in grains (Krzysztof et al., 1982; Rouau et al., 1997).

### Lignans

Lignans are mostly found in plant seeds and are precursors to phytoestrogens. They are a class of secondary plant metabolites. There is a growing interest in lignans in recent years due to their strong bioactivities in antioxidation, anti-inflammation and neuroprotection (Saleem et al., 2005; Teponno et al., 2016). The richest dietary source of lignans are linseeds (Thompson et al., 1991).

### Stilbenes

Stilbenes are poorly present in the human diet. The most known and studied is resveratrol, for which anticarcinogenic effects have been shown in medicinal plants screening. Resveratrol is found in wine at a very low quantity (Bertelli et al., 1998; Bhat and Pezzuto, 2002; Vitrac et al., 2005).

## Polyphenols modulate Aβ production, oligomerization, and clearance

Senile plaques are mainly composed of β-Amyloid protein (Aβ; Masters et al., 1985) that results from proteolysis of the amyloid precursor protein (APP) by the enzymes β-secretase (BACE) and γ-secretase through amyloidogenic pathway. The non-amyloidogenic process is initiated by α-secretase rather than BACE leading to the formation of soluble APPα and C-terminus fragments and preventing Aβ generation. Both enzymes compete in APP proteolysis and their activities strongly affect Aβ production.

Several studies have demonstrated that polyphenols can modulate Aβ production by either increasing α-secretase activity or inhibiting BACE. (−)-Epicatechin, epigallocatechin, epigallocatechin-3-gallate (EGCG) and curcumin are potent inhibitors of amyloidogenic processing (Wang X et al., 2014; Cox et al., 2015; Guo et al., 2017). *In vitro* experiments conducted in neuronal cell line expressing human APP showed that treatment with EGCG significantly decreased Aβ production (Rezai-Zadeh et al., 2005). These results have been reproduced *in vivo*, where intraperitoneal injection of epigallocatechin-3-gallate in Tg2576 mouse model of AD decreased Aβ levels and favored the non-amyloidogenic α-secretase mediated pathway (Rezai-Zadeh et al., 2005). Another study demonstrated that curcumin treatment increases α-secretase activity (Narasingappa et al., 2012). Curcuminoids and epigallocatechin-3-gallate treatment inhibit BACE activity in neuronal cells (Wang X et al., 2014). While EGCG alone (Cheng et al., 2012) failed to abolish BACE activity *in vivo*, in combination with ferulic acid (FA), a BACE modulator (Mori et al., 2013), epigallocatechin-3-gallate could block BACE activity in APP/PS1 AD mice and reduced amyloidosis and improved cognitive function (Mori et al., 2019). The flavones apigenin (Zhao et al., 2013) and nobiletin (Nakajima et al., 2015) were also shown to significantly reduce soluble and insoluble Aβ as well Aβ deposits in the brain in AD mice (Onozuka et al., 2008). Similarly, chronic administration of the flavone baicalein decreases Aβ production (Zhang et al., 2013). Quercetin (3,5,7,3′,4′-pentahydroxyflavone) is a dietary flavonol widely distributed in plants, fruits and vegetables, and it is also effective at modulating contents of soluble and insoluble Aβ in the brain (Sabogal-Guaqueta et al., 2015; Moreno et al., 2017). Altogether, these results show that select polyphenols can modulate α-secretase or BACE activities and reduce Aβ production both *in vitro* and *in vivo*, however, there has been very few research on mechanisms of action and how select polyphenols promote non-amyloidogenic or inhibit amyloidogenic processing of APP.

Aβ monomers can assemble into soluble and insoluble Aβ oligomers. Insoluble forms of Aβ mostly deposit into extracellular plaques, while the soluble oligomers are now considered the most toxic species in driving Aβ-mediated synaptic toxicity and neuronal death (Kumar et al., 2013). Polyphenols have been shown to prevent Aβ oligomerization or to remodel Aβ oligomers into nontoxic forms. Ehrnhoefer et al. showed that EGCG inhibits Aβ fibrillogenesis leading to unstructured Aβ oligomers. It promotes the assembly of newly formed oligomers into smaller and amorphous nontoxic protein aggregates (Ehrnhoefer et al., 2008). Another group showed (−)-epigallocatechin-3-gallate could bind to preformed fibrils or large oligomers and remodel them into less toxic assemblies (Bieschke et al., 2010). Curcumin can substantially block Aβ oligomerization in a dose dependent manner (Yang et al., 2005; Reinke and Gestwicki, 2007) and it is able to inhibit fibril formation and destabilize preexisting fibrils (Doytchinova et al., 2020). Resveratrol does not prevent Aβ oligomerization, however it can reduce Aβ cytotoxicity by remodeling the oligomers into nontoxic forms (Feng et al., 2009; Ladiwala et al., 2010; Fu et al., 2014). Wang et al., demonstrated that moderate red wine consumption could reduce Aβ aggregation, and improved cognitive function when administered to AD mice (Wang et al., 2006). The same group investigated the specific compounds responsible for Aβ-lowering activity and demonstrated that dietary supplementation with grape seed polyphenolic extract (GSPE), largely composed of catechin and epicatechin monomer, oligomer and polymer, significantly attenuated the development of AD-type Aβ-related cognitive deterioration (Wang et al., 2008). Further investigation revealed that GSPE is a potent inhibitor for the oligomerization of Aβ peptides. These observations demonstrate that by modulating or remodeling of Aβ oligomers, polyphenols can interfere with the formation of soluble toxic forms of Aβ that are responsible for AD-associated neuronal damages.

Polyphenols can also reduce Aβ pathology by enhancing Aβ clearance. For example, resveratrol was shown to facilitate Aβ clearance *in vitro* (Marambaud et al., 2005; Vingtdeux et al., 2010) but the mechanism remains poorly understood. Several hypothesis have been proposed, for example resveratrol may promote intracellular degradation of Aβ *via* mechanism that involves the autophage and lysosome (Marambaud et al., 2005). Resveratrol may also stimulate the brain insulin-degrading enzyme activity (Rege et al., 2015) which in return will degrade Aβ thereby facilitating Aβ clearance.

## Polyphenols modulate Tau phosphorylation

Aberrant aggregation of microtubule-associated protein Tau is another contributor to AD pathology. Tau phosphorylation regulates its ability to bind microtubules. Hyper phosphorylated Tau forms paired helical filaments (PHF) and neurofibrillary tangle (NTF) inclusions that not only alter the cytoskeletal and associated transport system, but also affect cellular signaling and mitochondrial function (Johnson et al., 2016; Bejanin et al., 2017; Kametani and Hasegawa, 2018).

Tau phosphorylation in neuronal cells is regulated by the balance of the dephosphorylation catalyzed mainly by phosphatase 2A (PP2A; Gong et al., 1993) and phosphorylation catalyzed by cdk5, GSK-3β, PKA and other kinases (Medina et al., 2011; Cavallini et al., 2013). Select polyphenols can modulate Tau hyperphosphorylation and subsequent NFTs formation through inhibiting AD-tau kinases or promoting PP2A. Resveratrol has been shown to inhibit the hyperphosphorylation of Tau (He et al., 2016); additionally, in the senescence accelerated mice P8 (SAMP8), resveratrol inhibits Ser^396^ Tau phosphorylation by GSK-3β (Porquet et al., 2013). Resveratrol can also modulate Tau hyperphosphorylation by increasing PP2A activity, which leads to Tau dephosphorylation (Schweiger et al., 2017). Similarly, in an okaidic acid-injection model for AD, curcumin treatment inhibited Tau hyperphosphorylation through activation of GSK-3β pathway (Wang et al., 2019). Oral GSPE supplementation is also effective in significantly modulating Tau-mediated pathogenic phenotypes, including Tau hyperphosphorylation, misfolding into fibrillar polymers and subsequently aggregation into AD-type NFT in various tauopathy mouse models (Ho et al., 2009; Wang et al., 2010; Ksiezak-Reding et al., 2012; Santa-Maria et al., 2012). GSPE is largely composed of proanthocyanidin (PAC) catechin and epicatechin in monomeric, oligomeic and polymeric forms. Bioavailability studies conducted in rats by Ferruzzi et al. (2009) demonstrated that methylated and glucuronidated catechin and epicatechin can be found in the plasm following oral administration of GSPE. Moreover, they also reported that following single oral dosing, these polyphenol metabolites were not found in the brain, however, following repeated dosing, these metabolites could be detected in the brain (Ferruzzi et al., 2009). Similar studies were conducted in AD mouse showing that catechin and epicatechin metabolites can only be found in the brain of the mouse fed with monomeric fraction of the GSPE, but not the polymeric fraction of the GSPE. Moreover, they reported that only the monomeric fraction were effective in reducing amyloid neuropathology and improving cognitive function in AD mice (Wang et al., 2012).

Biophysical studies demonstrated that polyphenols may structurally change Tau protein and prevent its self-association. For example, in the presence of arachidonic acid, Tau self-assembles into β-sheet containing filaments, but in the presence of curcumin, arachidonic acid-mediated filament formation is abolished (Rane et al., 2017). Similarly, epicatechin-3-gallate, myricetin(Taniguchi et al., 2005) and rosmarinic acid could also inhibit Tau β-sheet formation (Cornejo et al., 2017).

## Polyphenols modulate AD-associated oxidative stress

The brains of AD patients show significant oxidative stress-associated damage including protein oxidation, lipid peroxidation, DNA damage suggesting the imbalance of free radical generation and antioxidant activity in the brain (Christen, 2000). In AD brain, the main sources of oxidative stress are from the free radicals generated from mitochondria and redox-active metals. Lipid peroxidation occurs when these oxidants attack polyunsaturated fatty acids (Ramana et al., 2014). Free radical-induced lipid peroxidation is widespread in AD brain. Reactive oxygen species (ROS) can also attack amino acid side chains or the protein backbones and generate protein carbonyl derivatives (Butterfield and Stadtman, 1997). Protein carbonyl content was found to be significantly increased in the hippocampus and inferior parietal lobule in AD subjects comparing to normal controls (Hensley et al., 1995; Aksenov et al., 2001). ROS-induced oxidation of key enzymes or structural proteins can significantly impair their cellular function leading to neurodegeneration and cell death (Hensley et al., 1995; Butterfield and Stadtman, 1997; Aksenov et al., 2001). ROS can also leading to base alteration, single and double strand breaks or DNA-protein crosslinkings (Sohal and Weindruch, 1996; DNA Oxidation in Alzheimer's Disease, 2006).

The therapeutic efficacy of flavonoids is historically attributed to their antioxidant potency and natural free radical scavenging properties (Mercer et al., 2005). Chronic administration of nobiletin to AD mice for 2–3 months significantly reduced brain ROS (Nakajima et al., 2015) and other oxidative stress markers (Nakajima et al., 2013). Quercetin has been shown to be strongly effective at scavenging free radicals and preventing oxidant-induced apoptosis (Rice-Evans et al., 1995; Heijnen et al., 2002; Choi et al., 2003). In addition to its high oxygen radical scavenging properties, quercetin also has the ability to inhibit lipid peroxidation (Fiorani et al., 2010) and to chelate iron and other metal ions that could be detrimental to the brain (Rice-Evans et al., 1995; Salganik, 2001). Chronic administration of the flavone apigenin to an AD mouse model induced a significant decrease of oxidative stress accompanied by increased superoxide dismutase and glutathione peroxidase activities (Rice-Evans et al., 1995; Fiorani et al., 2010; Zhao et al., 2013). Curcumin is another strong antioxidant and can effectively stabilize ROS (Basnet and Skalko-Basnet, 2011). It acts on the inner membrane of mitochondria, facilitating their depolarization, thus preventing the formation of ROS (Zhu et al., 2004). It was also shown to stop the free radical proliferation when administered intravenously to rodent (Jiang et al., 2007). Resveratrol can increase the superoxide dismutase enzyme activity hence reduce ROS formation *in vivo* (Chen et al., 2016). *In vivo* studies have shown that catechin and epicatechin can prevent ROS formation and lipid peroxidation in AD models. A single oral dose of epicatechin could effectively prevent Aβ-mediated lipid peroxidation and ROS formation in hippocampal formation in rats (Cuevas et al., 2009). Blueberry is rich in anthocyanins and proanthocyanidins. Blueberry extract was shown to be able to increase redox buffer glutathione and protect amyloid toxicity through inhibition of MAP kinase and CREB-mediated ROS signaling (Brewer et al., 2010). In humans, it was shown that blueberry supplementation protects against cognitive decline in people with high risk for developing dementia (Krikorian et al., 2022).

## Polyphenols modulate AD-associated inflammation

There is increasing consensus that immunological perturbations are major contributors to AD pathogenesis. This is supported by the genome-wide association studies linking myeloid cell-specific genes, such as TYROBP, TREM2 and CD33, with late-onset AD (LOAD). The phenomenon called neuroinflammation is a critical factor in AD pathogenesis. While the exact role of inflammation in AD remains to be investigated, it has been suggested that acute and systemic inflammation, manifested by microgliosis and astrogliosis, can accelerate AD progression and worsen cognitive impairments (Holmes et al., 2009).

Many dietary polyphenols have demonstrated their anti-inflammatory activities both *in vitro* and *in vivo*. Among these, curcumin and resveratrol are the most studied molecules for their potential application in AD treatment. Curcumin was shown to be able to block NF-kappa B action and associated inflammation cascade (Singh and Aggarwal, 1995; Hackler et al., 2016). In addition, curcumin also has the ability to inhibit Aβ-induced pro-inflammatory cytokines and chemokines release (Sundaram et al., 2017). Resveratrol attenuates Aβ-mediated microglia inflammation through inhibition of the TLR4/NF-κB and/or NLRP3 and STAT signaling pathway (Capiralla et al., 2012; Feng and Zhang, 2019). Resveratrol can also activate SIRT1 both *in vitro* and *in vivo* (Herskovits and Guarente, 2014; Favero et al., 2018). SIRT1 is a histone deacetylase that can epigenetically reprogram inflammation. In animal models, resveratrol treatment improved spatial memory, reduced neuroinflammation and increased neurotrophins in the brain of AD mice (Gong et al., 2010; Sun et al., 2019; Broderick et al., 2020). In humans, resveratrol was shown to modulate neuroinflammation and induce adaptive immunity in patients with AD (Moussa et al., 2017). Subjects with mild–moderate AD treated with synthetic resveratrol showed significant decrease of MMP9 in the cerebral spinal fluid (CSF). MMP9 is a protein that interferes with the blood brain barrier (BBB) function. The decrease of CSF MMP9 in AD suggest that resveratrol may mitigate inflammatory responses in the brain by reducing the permeability of CNS and lower infiltration of leukocytes and other inflammatory agents into the brain. Other polyphenols such as flavonoids fisetin, quercetin and luteolin were also shown to decrease inflammation in different AD mouse models and reduce astrogliosis and microgliosis (Sharma et al., 2007; Currais et al., 2014; Currais et al., 2018). Blueberry and its extract have also been shown to be able to inhibit amyloid-mediated microglia activation through attenuation of p44/42 MAPK signaling both *in vitro* and *in vivo* mouse model (Joseph et al., 2003; Zhu et al., 2008).

## Polyphenols modulate synaptic function and memory

Selective polyphenols also showed promising effects in rescuing cognitive function in transgenic AD mouse models. For example, old 5xFAD mice chronically treated with 7,8-dihydroxyflavone (7,8-DHF) reduced synapse loss in the brain and performed better at the working memory Y maze test (Devi and Ohno, 2012; Zhang et al., 2014). Oral administration of apigenin led to increased activation of ERK/CREB signaling and learning and memory improvement in 2xFAD mice (Zhao et al., 2013). Learning and memory were improved in both the 1 × FAD and 3xFAD mouse models of AD following treatment with nobiletin (Onozuka et al., 2008; Nakajima et al., 2015). Mice treated with fisetin showed increased activation of ERK/MAPK signaling, increased expression of synaptic proteins and improved cognitive function (Currais et al., 2014; Ahmad et al., 2017; Currais et al., 2018). Flavonoid rutin, a quercetin molecule with the addition of disaccharide rutinose, was also found to be effective in improving cognitive function through increased expression of brain derived neurotrophic factor (BDNF) in rats injected with Aβ (Moghbelinejad et al., 2014). Green tea contains high levels of EGCG can prevent the loss of synaptic proteins and cognitive impairments in a 1xFAD mouse model (Walker et al., 2015). On the same note, anthocyanins were also found to be able to reduce synaptic protein loss and improve memory function (Ali et al., 2017; Kim et al., 2017). Anthocyanin from grape juice was shown to rescue oligomeric Aβ-induced long term potentiation (LTP) deficit in hippocampal slices (Wang et al., 2014a). Wang et al. explored the effect of cocoa flavanols on AD pathogenesis (Wang et al., 2014b). In their study they showed that catechin and epicatechin enriched cocoa extracts interfered with Aβ oligomerization and prevented synaptic deficits. They demonstrated that application of cocoa extracts on mice hippocampal slices could prevent Aβ-induced LTP deficit (Wang et al., 2014b). Cocoa was also shown to prevent Aβ oligomer-induced neurite dystrophy by activating BDNF in neuronal cultures (Cimini et al., 2013). These observations have also been corroborated by clinical studies demonstrating that cocoa flavanols enhance the dentate gyrus function and reduces cognitive decline in humans (Crews et al., 2008; Desideri et al., 2012; Scholey and Owen, 2013; Brickman et al., 2014). Blueberry was shown to improve memory function in APP/PS1 AD mice through increase of ERK signaling and neural sphingomyelin-specific phospholipase C activity (Joseph et al., 2003).

## Polyphenols and gut microbiome

The biological activity of dietary polyphenols largely depends on the bioavailability of the bioactive forms of the parent compounds in the target organs. Once ingested, polyphenols are absorbed and metabolized first in the gastrointestinal tract and then are further modified in the liver through glucuronidation, sulfonation, or methylation, before entering the blood stream. The biological activities of their metabolites can be very different from the parent compounds. For example, Serra et al. explored this relationship between dietary polyphenols and gut metabolism using an anthocyanin-rich extract obtained from Portuguese blueberries and a simulated gastrointestinal digestion process. Both the digested and non-digested extracts displayed different chemical compositions and had different effects on neuroinflammation (Serra et al., 2020). Gut microbiome is receiving increased attention due to their potential role in health and disease (Durack and Lynch, 2019). Recent studies have demonstrated strong links between polyphenol metabolism and the gut microbiome (Hervert-Hernández and Goñi, 2011; Fraga et al., 2019). The gut microbiota can influence the process and metabolism of polyphenols, which may influence the production and diversity of polyphenol metabolites; Polyphenols have the ability to influence the intestinal environment, which allows them to modulate the composition of gut microbiome (De Bruyne et al., 2019). Moreover, there is also bidirectional communications between gut microbiota and the central nervous system, the so-called gut-brain axis and currently, the gut-brain axis is one of the favorable targets for therapeutic treatment of neurodegenerative disorders including AD due to their bidirectional interactions that may affect brain function (Carabotti et al., 2015; Reddy et al., 2020). Curcumin, for example, exhibits beneficial effects against AD despite having limited blood–brain barrier penetration. It is postulated that curcumin becomes a more effective neuroprotective agent after undergoing metabolism by gut microbial and its interaction with the gut-brain axis also allows it to react indirectly with the CNS and exerts its neuroprotective activity (Di Meo et al., 2019; Reddy et al., 2020).

## Conclusion

Pathological mechanisms involved in Alzheimer’s pathogenesis include both Aβ and Tau toxicity. Therapeutic strategies aiming at targeting one or the other continue to fail in clinical trials. Polyphenols offer a new approach that can simultaneously target Aβ, Tau, neuroinflammation and oxidative stress, which could lead to better outcomes.

Dietary factors and diet composition can play a critical role in AD prevention. Our review lists few of the numerous polyphenols, considered as confirmed or promising therapeutic candidates due to their potent anti-inflammatory, antioxidant properties and AD-disease modifying activities (Table 1). AD is a multifactorial disease and the current lackluster performance of clinical studies is, in part, due to the prevailing approach targeting individual pathogenic mechanisms. Most of the polyphenolic compounds have exhibited pleiotropic bioactivities (Table 1) which may have advantages over conventional pharmaceutical drugs for the treatment of AD. In spite of their multi-targeting features, clinical development of polyphenols for AD is hampered by their poor absorption and limited brain bioavailability. Moreover, most of the available polyphenol metabolite forms, following digestive and hepatic activity, may not have the same biological activity as the native compound. Therefore, the *in vitro* biological activities of “parental” polyphenol forms may not relevant to biological activities *in vivo*, which is the ultimate arbitrator of therapeutic benefit. Future advancement of polyphenols in AD prevention and/or treatment will largely rely on the development of select polyphenols or their derivatives with better brain bioavailability while preserving their multi-targeting bioactivities.

**Table 1:** Role of polyphenols in modulating AD-type neuropathology.

Polyohenol	Activity	Mechanism	Model	Dose	Reference
Epicatechin	Aβ↓	BACE↓	TASTPM	15 mg/day	Cox et al., 2015
	ROS↓	-	Aβ injected rat	30 mg/kg	Cuevas et al., 2009
EGCG	Aβ↓	α-secretase ↑	Tg2576	20 mg/kg	Rezai-Zadeh et al., 2005
	Aβ↓/memory↑	BACE↓	SAMP8	15 mg/kg	Guo et al., 2017
	Aβ fibril↓	-	*in vitro*	-	Ehrnhoefer et al., 2008; Bieschke et al., 2010
Curcumin	Aβ ↓	BACE↓	Drosophila Melanogster	1 mM	Wang et al., 2014
	Aβ oligomer↓	Aβ aggregation↓	Tg2576	25 mg/kg	Yang et al., 2005
	Tau phosphorylation↓	GSK-3β↑	okadaic acid AD model	10 μg i.p.	Wang et al., 2019
	neuroinflammation↓	NF-κB↓	*in vitro*	-	Singh and Aggarwal, 1995
	neuroinflammation↓/Memory↑	CDK5↓	p25Tg	0.8 g/kg	Sundaram et al., 2017
Apigenin	Aβ↓/ROS↓/memory↑	BACE↓	APP/PS1	40 mg/kg	Zhao et al., 2013
	ROS↓	SOD↑/GPx↑	*in vitro*	-	Rice-Evans et al., 1995; Fiorani et al., 2010
Nobiletin	Aβ↓/ROS↓/memory↑	-	3xTg	30 mg/kg	Nakajima et al., 2015
	Aβ↓/memory↑	ERK phosphorylation↑	APP-SL 7–5 Tg	10 mg/kg i.p.	Onozuka et al., 2008
	Tau phosphorylation↓/ROS↓/memory↑	-	SAMP8	10–50 mg/kg	Nakajima et al., 2013
Quercetin	Aβ↓/memory↑	BACE↓	3xTg	25 mg/kg	Sabogal-Guaqueta et al., 2015
	neuroinflammation↓/memory↑	-	SAMP8	25 mg/kg	Moreno et al., 2017
	ROS↓	-	*in vitro*	-	Choi et al., 2003; Heijnen et al., 2002; Rice-Evans et al., 1995; Fiorani et al., 2010
Resveratrol	Aβ oligomer toxicity ↓	-	*in vitro*	-	Feng et al., 2009; Ladiwala et al., 2010; Fu et al., 2014
	Aβ ↓	-	*in vitro*	-	Marambaud et al., 2005
	Aβ clearance ↑	AMPK/mTOR autophage	*APP/PS1*	350 mg/kg	Vingtdeux et al., 2010
	Aβ clearance ↑	IDE↑	*in vitro*	-	Rege et al., 2015
	Tau phosphorylation↓/memory↑	ADAM-10↑/GSK-3β↑/CDK5↓	SAMP8	1 g/kg	Porquet et al., 2013
	neuroinflammation↓/Aβ oligomer↓/memory↑	NF-κB↓	SAMP8	4 g/kg	Broderick et al., 2020; Gong et al., 2010; Sun et al., 2019
Fisetin	neuroinflammation↓/ROS↓/memory↑	CDK5↓/SAPK/JNK↓	SAMP8	25 mg/kg	Currais et al., 2018
	neuroinflammation↓/memory↑	CDK5↓	APPswe/PS1dE9	25 mg/kg	Currais et al., 2014
GSPE	Aβ oligomer↓/memory↑	Aβ aggregation↓	Tg2576	200 mg/kg	Wang et al., 2008
	tau aggregation↓	-	*in vitro*	-	Ho et al., 2009; Ksiezak-Reding et al., 2012
	Tau phosphorylation↓	ERK1/2↓	TMHT	200 mg/kg	Wang et al., 2010
	Tau phosphorylation↓/tau aggregation↓/motor function↑	-	*JNPL3*	150 mg/kg	Santa-Maria et al., 2012

EGCG: epigallocatechin-3-gallate; GSPE: grape seed polyphenolic extract.

## Author contributions

FE, FC, LH, and JuW wrote, edited, and reviewed the manuscript. H-YL, CY, and JeW reviewed and edited the manuscript. All authors contributed to the article and approved the submitted version.

## Funding

Funding was provided by the P50 AT008661-01 from the National Center for complementary and Integrative Health (NCCIH) and the Office of Dietary Supplements (ODS). In addition, JuW holds a position in the Research and Development unit of the Basic and Biomedical Research and Training Program at the James J. Peters Veterans Affairs Medical Center.

## Conflict of interest

The authors declare that the research was conducted in the absence of any commercial or financial relationships that could be construed as a potential conflict of interest.

## Publisher’s note

All claims expressed in this article are solely those of the authors and do not necessarily represent those of their affiliated organizations, or those of the publisher, the editors and the reviewers. Any product that may be evaluated in this article, or claim that may be made by its manufacturer, is not guaranteed or endorsed by the publisher.
